# Living in Biological Darkness II: Impact of Winter Habitual Daytime Light on Night‐Time Sleep

**DOI:** 10.1111/ejn.16647

**Published:** 2025-01-20

**Authors:** Claudia Nowozin, Amely Wahnschaffe, Jan de Zeeuw, Alexandra Papakonstantinou, Sven Hädel, Andrea Rodenbeck, Frederik Bes, Dieter Kunz

**Affiliations:** ^1^ Institute of Physiology, Sleep Research & Clinical Chronobiology Charité – Universitätsmedizin Berlin Berlin Germany; ^2^ Clinic Sleep & Chronomedicine St. Hedwig‐Krankenhaus Berlin Germany; ^3^ Sleep Clinic Evangelisches Krankenhaus Goettingen‐Weende Göttingen Germany

**Keywords:** biological darkness, depression marker, habitual daytime lighting, REM latency, sleep, SWS

## Abstract

Timing and architecture of sleep are co‐driven by circadian rhythms modulated by their major *Zeitgeber* light and darkness. In a natural environment, one is exposed to 3.000 lx (cloudy winter sky) to 100.000 lx (bright sunny sky). The aim of the study was to assess (1) habitual daytime light exposure in urban winter and (2) impact of daytime urban light on objective night‐time sleep. Eleven healthy participants (mean age ± SD: 25.4 ± 2.8 years; 6 male) wore eyeglass frames continuously recording daytime illuminance levels vertically to the eye by mounted sensors (range: 1–40.000 lx) during four consecutive days in winter 2008 in Berlin, Germany. In‐lab polysomnography was performed over two nights in nine participants. Median light exposure over 4 days was the following: full day 7:00–19:00 h: 23 lx (12–37 lx); morning 7:00–11:00 h: 81 lx (19–201 lx); midday 11:00–15:00 h: 68 lx (19–164 lx); afternoon 15:00–19:00 h: 22 lx (6–58 lx), resulting in only 36 min > 500 lx per day. Timing of daytime light intensity was significantly associated with subsequent sleep: lower midday illuminance with shorter REM latency (Rho = 0.817; *p* = 0.049) and earlier REM polarity (less prevalence of REM at end‐of‐sleep; Rho = 0.817; *p* = 0.049). Humans, living in an urban environment, appear to be exposed to extremely low light levels, which we named as ‘*Living in Biological Darkness*’. Most fascinating, physiology seems to adapt and responds to variation in light intensity on such low levels. Interestingly, the observed changes in sleep architecture with low light levels are reminiscent of those suspected to constitute biological markers of depression some 40–50 years ago.

## Graphical abstract

Light perceived by healthy subjects during winter in Berlin, measured 4 days vertical at the eye, was extremely low (< 200 lx for 90% of the day; < 20 lx for about 30% of the day). Individual variation on this low daytime light levels were associated with differences in sleep architecture including a depression‐like pattern in subjects with the lowest light levels. Thus, human physiology seems to adapt to low artificial light levels we name ‘*Living in Biological Darkness*’.

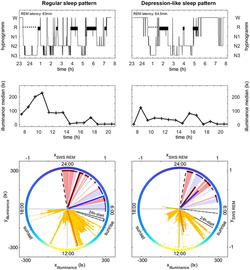

**Abbreviations**
AASM: American Academy of Sleep Medicine
DLMO: dim light melatonin onset
EEG: electroencephalogram
NREM: non‐rapid eye movement sleep
N1: NREM sleep stage 1
N2: NREM sleep stage 2
N3: NREM sleep stage 3
PSG: polysomnography
PSQI: the Pittsburgh Sleep Quality Index
REM: rapid eye movement
REMS: REM sleep
SAD: seasonal affective disorder
SPT: sleep period time
SWS: slow wave sleep
TST: total sleep time
WHO: World Health Organization

*… give me new noise, give me new affection, strange new toys, from another world, I need to see more than just three dimensions, stranger than fiction, faster than light*. Steven Brown

What a coincidence. These lines in the song What Use! written by the creative head of Tuxedomoon, Steven Brown, were among my all‐time favourites long before I happened to meet another Steven Brown some 20 years ago. He came with Achim Kramer, asked for a cooperation in a project on molecular mechanisms in chronotypes. ‘*Molecular mechanisms in chronotypes?!?*’ There it was: ‘*stranger than fiction, faster than light*.’

Steven was one of the few driven by a utopia to follow, provided with a bright and clear mind. Killed in a plane crash in Newfoundland: Startling, unbearable, but surprising? What a way to go.

Dieter Kunz

## 1 Introduction

Major depression—a common and heterogenous illness—was ranked by the World Health Organization (WHO) as one of the top three burden of disease (WHO 2008). Because etiopathogenesis and pathophysiology are still widely unknown, diagnosis relies on clinical examination and subjective evaluation of depressive symptoms (Malhi and Mann 2018).

Some 40 years ago, chronobiology was an essential topic in the emerging field of biological psychiatry (Wehr and Goodwin 1983). Realization of a circadian timing system existing in humans (Aschoff 1993; Pittendrigh 1993; Wehr et al. 1993) provided an insight into the physiological basis of various symptoms in psychiatric disturbances, such as seasonal variation of mood, drive and sleep length in seasonal affective disorder (SAD) (Rosenthal et al. 1984), diminished circadian rest‐activity‐rhythm in demented patients (Swaab et al. 2002) or circadian variation in mood of depressed patients resulting in morning blues and evening euthymia (Kripke et al. 1978). Most important, circadian‐influenced sleep parameters such as shortened REM sleep (REMS) latency and shift of slow‐wave sleep (SWS) to later sleep times were suggested as biological markers of depression (Gillin et al. 1979; Kupfer et al. 1977). However, it later turned out that these sleep peculiarities were nonspecific and that improvement in symptoms was not accompanied by changes in these characteristics (Kupfer, Bulik, and Grochocinski 1984). The field of psychiatry lost interest. Sleep and circadian factors were considered symptoms, but not causal factors or biomarkers in mental illnesses anymore (World Health Organization 1993).

The sleep–wake cycle of the diurnal human species is characterized by daytime wake and night‐time sleep preferences. Human physiology identifies the time of day and season based on light or its absence, resulting in the synchronization to environmental time (Czeisler et al. 1995), which is one core interest in the field of chronomedicine. As light and darkness were early on singled out as *Zeitgeber* to synchronize endogenous circadian rhythms with the environmental 24‐h day (Winget et al. 1970), light therapy was applied as a therapeutic agent to stabilize disturbed circadian rhythms in psychiatric illnesses. Although substantial efforts were made, the established benefits of light therapy with respect to individual diagnosis and therapy remained limited to disturbances such as SAD (Rosenthal et al. 1984) and night‐time activity in dementia (Van Someren et al. 1997). This may have added to a loss of interest in chronobiology's stake in psychiatry.

However, over the past two decades, research provided new insights in structures and mechanisms involved in circadian aspects. A new photopigment was identified in the retina and later named melanopsin. Melanopsin appeared to be sensitive to blue spectrum light and—among several other actions—informs the brain and body of the time and length of the day (Brainard et al. 2001; Thapan, Arendt, and Skene 2001). In healthy participants, physiology adapts to environmental light levels, causing a sensitization to light—measured in saliva melatonin secretion—after days, or even hours of reduced light levels (Chang, Scheer, and Czeisler 2011; Hébert, Martin, and Eastman 2002). This phenomenon called ‘light history’ suggests that daytime light impacts physiological processes during subsequent nights. Absence of light in the evening causes melatonin release, the signal of darkness, signalling environmental night to almost every cell in the human body (Pevet and Challet 2011). The sleep state predominantly influenced by the circadian timing system is REMS (Bes et al. 1996). Low individual evening/night‐time melatonin is associated with reduced REMS (Mahlberg and Kunz 2007). Furthermore, when exogenous melatonin was given to patients with low REMS, time spent in REMS increased and REMS stabilized (Kunz et al. 2004). These phenomena imply that habitual low daytime light, which is common in winter, indoors and urban environment, may have effects on night‐time sleep. Effects of such low daytime light on subsequent sleep have seldom been studied.

The aim of the study was to assess (1) habitual daytime light exposure in urban winter in a group of healthy subjects and (2) impact of such daytime urban light on objective night‐time sleep. This includes REMS latency, REMS density, REMS polarity and nocturnal distribution of SWS, all once considered as biomarkers of depression (Kupfer, Bulik, and Grochocinski 1984).

## 2 Materials and Methods

The study was performed in November 14th–December 8th 2008 and had been designed to describe the association of habitual daytime light measured at the eye with the circadian influenced sleep parameters in the following night, modulated by differential light on four evenings. Here, we only report the effects of daytime light variation on night‐time sleep for the day when participants received the dim light control condition in the evening. The order of the 4 days with evening light exposure was randomized.

### 2.1 Participants

Eleven healthy participants (age 25.4 ± 2.8 years; mean ± SD; 6 male, 5 female) were recruited via bulletins at universities in Berlin, Germany. Inclusion criteria were as follows: age 20–35 years; habitual bed‐time 22:00–24:00 h (22:58 h ± 27 min; mean ± SD). Exclusion criteria were as follows: poor sleep (PSQI < 5) (Buysse et al. 1989); extreme chronotype (49.6 ± 15.9; mean ± SD) (Horne and Östberg 1976); chronic medical or mental disease; medication; history of drug or alcohol abuse; regular nightshift during past 12 months; travelling more than two time zones during previous 3 months. In the nine participants where a PSG was performed, the DLMO occurred on average (±SD) at 20:56 h (±00:30 h).

The week preceding the study, participants were required to keep regular bedtimes (23:00 h ± 30 min) and not take naps. This was controlled via actigraphy (Actiwatch, Cambridge Neurotechnology, UK) and sleep logs. Participants were instructed to abstain from caffeine after 14:00 h and alcohol all day long. They gave their informed written consent and received financial compensation. All study procedures were approved by the local Ethics Committee of the Charité‐Universitätsmedizin Berlin, Germany (EA2/015/07; 22.03.2007).

### 2.2 Study Design, Procedures

The data analysed here were collected in an intervention study with different evening light conditions performed November–December 2008 at Freie Universität, Institute of Physiology, Berlin. The influence of four evening light exposure conditions on sleep including one control condition with dim light only was examined. The melatonin suppression data of this experiment have been previously published in a study that combined data from multiple experiments (Nowozin et al. 2017).

**FIGURE 1:**
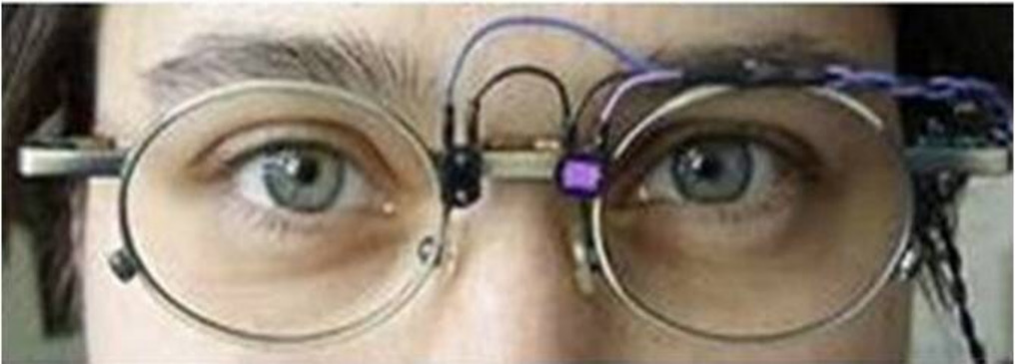
Glasses with mounted Luxblick sensors.

During daytime (07:00–19:00 h), participants followed their regular activities. Illuminance (lx) was continuously recorded with devices, mounted on the frames of glasses between the eyes and in the direction of gaze (Figure 1, Luxblick, Technical University in Ilmenau, Germany). Light sensors had a recording frequency of 1 Hz. Once in the laboratory (19:00 h), participants spent the time in dim light (< 5 lx, incandescent light source, < 1500 K) to minimize acute effects of evening light on subsequent sleep. They could perform self‐chosen activities like reading or talking. Devices with a lit screen (mobiles, tablets and laptops) were not allowed. During this time, they were prepared for the polysomnography (PSG), which started at 23:00 h. Saliva samples (Salivettes, Nümbrecht, Germany) were taken every 30 min, starting at 20:00 h until bedtime at 23:00 h. The collected samples were immediately stored at a maximum temperature of 4°C and frozen at −32°C within the following 12 h. Frozen saliva samples were sent to IBL (Hamburg, Germany) on dry ice, where saliva melatonin levels were determined with radio‐immunoassay.

Firstly, we analysed habitual light exposure over all 4 days of the study. Second, we analysed the effect of habitual light exposure on sleep in the subsequent PSG after the evening control condition (dim light only). PSG was performed according to AASM criteria (Iber et al. 2007) and started at 23:00 h in complete darkness (0 lx).

### 2.3 Data Processing and Analyses

The range of reliable measurements for the Luxblick devices was 1–40.000 lx (Wolf 2009). Taking into account the specifications of the Luxblick devices (Wolf 2009), missing data and artefacts were dealt with as follows. Because measurements below 1 lx were reported not to be reliably distinguishable from each other, yet could still be expected to be very low, we chose to set positive measurements < 1–1 lx to minimize missing data and to categorize negative values as artefacts. Similarly, precision was known to decrease with illuminances higher than 40.000 lx. So, if a measurement was > 40.000 lx, we set it to 40.000 lx. Since we analysed data medians, these deviations would have a negligible influence on the results. After cleaning raw data, a total average (±SD) of 87.2% (±9.1%) of the recorded data from all the 4 days was used for analysis. During the day before the dim light condition, the nine participants that were included in the analysis had 86.2% (±4.7%) of valid data. Continuous light exposures from 7:00 to 19:00 h were analysed. Time of maximal light exposure (based on 20 min bins) and dim light melatonin onset (DLMO) were included to assess associations with circadian phase shifts. Light data were binned in medians for three separate time slots with 4‐h length meant to represent morning (07:00–11:00 h), midday (11:00–15:00 h) and afternoon (15:00–19:00 h) hours.

### 2.4 Habitual Light Exposure

To describe habitual light exposure, the illuminance levels of the 4 days in the 11 participants were considered. Thus, medians, means, 10th and 90th percentiles were calculated as well as percentages of time (valid measurements) spent in low (< 20 lx), moderate (< 80 lx), medium (< 200 lx) and relatively high (< 500 lx) illuminances.

### 2.5 Associations Between Light Exposure and Subsequent Sleep

We calculated medians, means and percentages of illuminance levels for the single day with evening dim light in the same way as it is described above for the 4 days. For correlations with sleep parameters, we applied the median of light exposure during the assigned time frames. We chose medians instead of means, because we assumed that short bursts of high illuminance would have a relatively smaller impact on sleep than continuous exposure. These bursts would have high impact on the mean but less on the median. Due to technical problems, PSGs of two participants could not be used for analysis. Thus, PSG data of nine participants were finally included.

DLMO was determined as the time when melatonin concentrations reached a threshold of 10 pg/mL, applying the hockey‐stick method (Danilenko et al. 2014).

Sleep recordings were scored according to standard criteria (Iber et al. 2007), and data for slow wave sleep (SWS = non‐REM stage N3), REMS and non‐REM stage N2 were included in the analysis, expressed as percentage of sleep period time (SPT, time from sleep onset to end of sleep). Sleep onset was defined as the first 2 min of N2, possibly including N1, N3 or REM, but without Wake. Additionally, we included REMS latency, defined as the time from sleep onset to the first epoch of REMS.

### 2.6 Polarity of REMS and SWS

Because REM and SWS in healthy sleep are unevenly distributed over time, with long SWS episodes prevailing mainly early in sleep and long episodes of REMS rather towards the end of sleep, we quantified this phenomenon (‘polarity’) using mathematical tools. Therefore, we considered each scored 30‐s epoch within the SPT as a vector with standard length 1 and angle expressed by the respective clock time. We then placed those vectors in a 24‐h polar coordinate system, forming a representation of the hypnogram within a circular 24‐h sleep–wake continuum (Figure 3). Even if this may seem trivial, it is not, since this simple transformation now allows us to sum the vectors of similar sleep stages. Thus, the angle of the summed vector of all REMS epochs represents a centroid: a weighted sum, depending on the presence and clustering of REMS in the hypnogram, which corresponds to one specific point in time that we can use as an anchor point for REMS polarity. The strength of the polarity is expressed by the length of the summed vector. We proceeded in a similar way for SWS polarity.

### 2.7 Statistics

Since the data were not normally distributed (Kolmogorov‐Smirnoff: *p* < 0.05), all statistical analyses were performed with non‐parametrical tests (Spearman's Rho). The *p*‐values of the correlations were adjusted for multiple comparisons (i.e., the seven time frames of light exposure) using the Bonferroni‐Holm correction.

## 3 Results

### 3.1 Habitual Light Exposure

**FIGURE 2:**
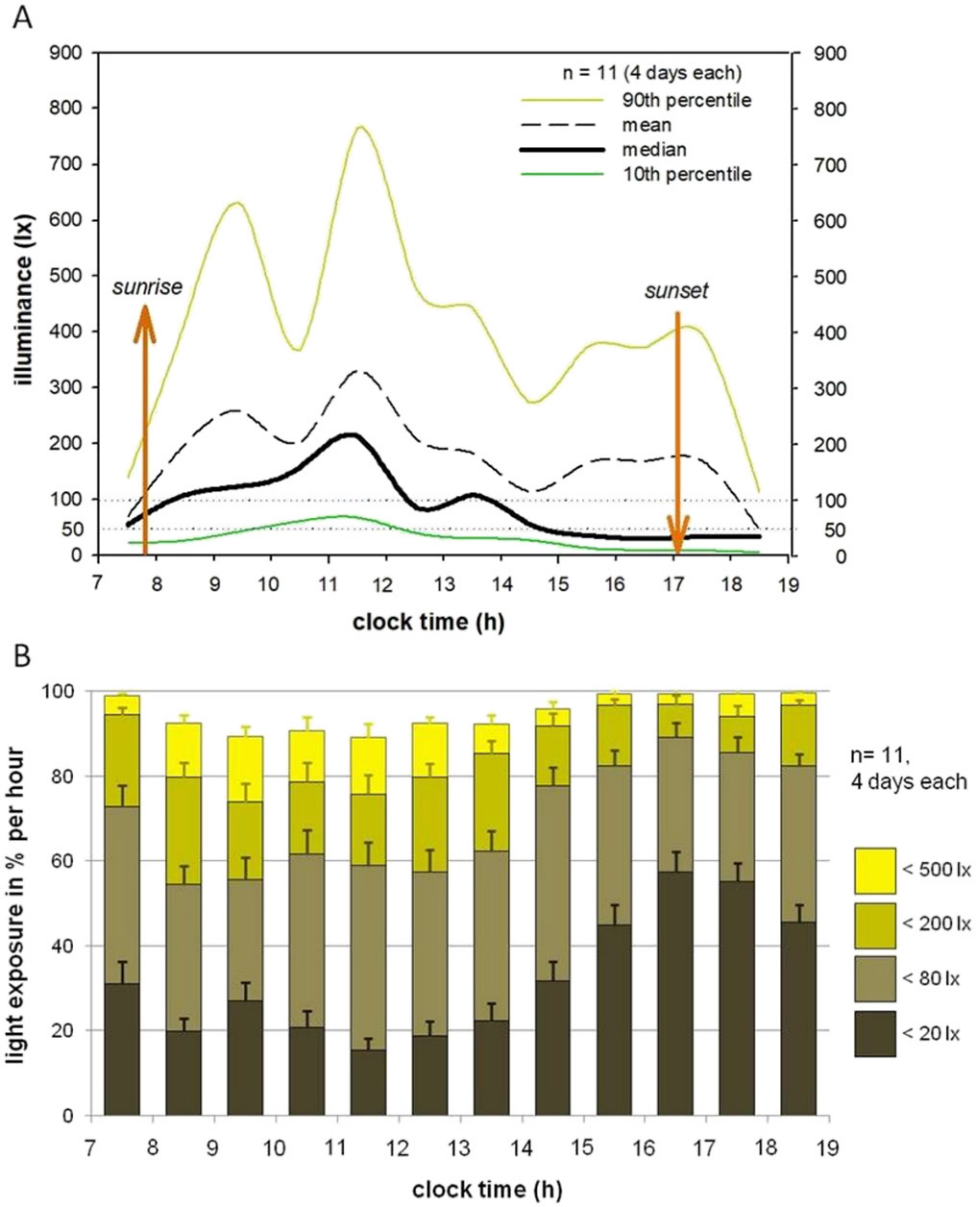
Light exposure during habitual daytime activities. Daytime illuminance during 4 days per participant (*n* = 11). (A) Group averages of 90th percentiles (dark yellow), means (dashed black), medians (bold black) and 10^th^ percentiles (dark green) of individual illuminances per hour. Smooth line represents dots of hourly means. Sunrise and sunset were derived from meteorological data (www.sunrise‐and‐sunset.com) and averaged for study duration (Berlin, Germany; 14th November – 8th December 2008; sunrise 7:27–8:04; sunset 15:51–16:13). (B) Data assigned to categories: < 20, < 80, < 200 and < 500 lx; expressed as mean percentage of time spent in these categories per hour; standard errors indicated by error bars.

Detailed descriptions of hourly daytime light exposure, averaged over 4 days for 11 participants are depicted in Table 1, Columns 2–7, and in Figure 2. Light exposure was highest in the morning, then became gradually lower, interrupted by a small noon peak and was lowest in the afternoon and evening. After 15:00 h, participants spent about half of their time in light levels below 20 lx and less than a fifth in light levels higher than 80 lx. The median amount of time (valid measurements per hour between 7:00 and 19:00 h; mean ± SD over all participants and days) spent in illuminance levels below 20 lx was 32.5% (±14.7%) of the day, 70.0% (±12.9%) below 80 lx, 87% (±9%) below 200 lx and 94.9% (±4.3%) below 500 lx. The latter indicates that participants spent on average only 36 min per day in illuminance levels of more than 500 lx.

**TABLE 1:** Descriptives of habitual light exposure.

Time of day	Over 4 days; *n* = 11						Day prior to PSG; *n* = 9					
	In lx		% of time				In lx		% of time			
	Median (range)	Mean (±SD)	< 20 lx Mean (±SD)	< 80 lx Mean (±SD)	< 200 lx Mean (±SD)	< 500 lx Mean (±SD)	Median (range)	Mean (±SD)	< 20 lx Mean (±SD)	< 80 lx Mean (±SD)	< 200 lx Mean (±SD)	< 500 lx Mean (±SD)
All day: 07:00–19:00 h	22.8 (12.2–37.1)	99.4 (±428.5)	32.5 (±14.7)	70.0 (±12.9)	87.0 (±9.0)	94.9 (±4.3)	40.7 (21.1–116.0)	49.8 (±29.4)	32.5 (±17.5)	72.0 (±14.1)	90.4 (±9.4)	96.5 (±3.7)
Morning: 07:00–11:00 h	81.1 (19.2–201.0)	238 (±669.6)	24.7 (±5.3)	61.1 (±8.4)	81.6 (±8.9)	92.8 (±4.3)	55.7 (19.2–201.0)	81.1 (±67.2)	23.1 (±6.3)	59.8 (±12.6)	81.2 (±9.0)	93.6 (±3.4)
Midday: 11:00–15:00 h	68.2 (19.4–164.0)	135.5 (±337.2)	22.1 (±7.1)	64.1 (±9.3)	83.1 (±7.0)	92.4 (±2.8)	50.0 (19.4–164.0)	68.2 (±50.1)	22.2 (±14.1)	69.5 (±5.7)	91.4 (±6.5)	96.2 (±3.6)
Afternoon: 15:00–19:00 h	21.8 (6.4–57.5)	38.7 (±64.5)	50.8 (±6.5)	84.8 (±3.2)	96.1 (±1.3)	99.4 (±0.1)	15.4 (6.4–57.5)	21.8 (±16.5)	52.3 (±9.9)	86.6 (±6.4)	98.5 (±0.3)	99.6 (±0.2)

*Note:* Median and mean light exposure based on individual medians per 4‐h bins over all 4 days (*n* = 11; Columns 2 and 3). Percentages of light exposure below certain illuminance levels per 4‐h time bin over all 4 days (Columns 4–7). Median and mean light exposure based on individual medians per 4‐h bins for the day before evening dim light only (*n* = 9; Columns 8 and 9). Percentages of light exposure below certain illuminance levels per 4‐h time bins for the day before evening dim light only (*n* = 9; Columns 10–13).

Median light exposure over medians of all participants and days during the entire day (7:00–19:00 h) was 23 lx (range: 12.2–37.1 lx); during morning hours (7:00–11:00 h), it was 81.1 lx (19.2–201 lx); during midday hours (11:00–15:00 h), it was 68 lx (19–164 lx); and during afternoon hours (15:00–19:00 h), it was 22 lx (6–58 lx).

### 3.2 Associations Between Light Exposure and Subsequent Sleep

Descriptive statistics for light and sleep data during the 1 day with evening dim light exposure are displayed in Table 1, Columns 8–13, and Table 2. Illuminance during that day was slightly lower, yet comparable with the data of the whole 4 days. The mean (±SD) time of maximal light exposure was 11:12 h (±02:43 h). Correlations between sleep stages and median light exposure are shown in Table 3.

**TABLE 2:** Polysomnography.

Sleep stages (n = 9)	Mean (±SD)
Sleep onset (hh:mm)	23:33 (±0:26)
Sleep offset (hh:mm)	7:07 (±0:29)
Midpoint of sleep (hh:mm)	3:13 (±0:14)
Sleep period time (SPT; min)	454.6 (±32.7)
Total sleep time (TST; min)	426.0 (±59.4)
Sleep efficiency (TST/SPT; %)	93.4 (±8.5)
Sleep latency (min)	26.4 (±23.8)
REMS latency (min)	71.0 (±11.8)
Sleep stage N1 (%)	4.3 (±1.1)
Sleep stage N2 (%)	41.8 (±7.3)
SWS (N3) (%)	24.2 (±7.3)
REMS (%)	23.1 (±4.7)

*Note:* Descriptives for sleep stages after habitual daylight (07:00–19:00 h) and evening dim light (19:00–23:00 h). Minutes (min) and percentage (%) are reported during/relative to sleep period time (SPT; *n* = 9).

Abbreviations: REMS = rapid eye movement sleep; SWS = slow wave sleep.

**TABLE 3:** Correlation of prior daytime illuminance and sleep parameters.

Time of day		N2 (min)	SWS (min)	SWS polarity		REMS (min)	REMS latency	REM polarity	
				Time	Strength			Time	Strength
Morning									
07:00–09:00 h	Rho	−0.017	−0.433	−0.100	−0.367	−0.183	−0.650	0.533	0.000
	*p*	0.999	0.999	0.999	0.999	0.999	0.406	0.973	0.999
09:00–11:00 h	Rho	0.017	0.083	−0.033	0.017	−0.100	0.150	0.167	−0.067
	*p*	0.999	0.999	0.999	0.999	0.999	0.999	0.999	0.999
Midday									
11:00–13:00 h	Rho	0.667	0.100	−0.400	−0.033	0.167	0.**817** *	0.**817** *	0.167
	*p*	0.350	0.999	0.999	0.999	0.999	0.**049**	0.**049**	0.999
13:00–15:00 h	Rho	0.100	0.783	0.150	0.683	−0.033	0.717	0.567	−0.017
	*p*	0.999	0.091	0.999	0.294	0.999	0.210	0.784	0.999
Afternoon									
15:00–17:00 h	Rho	**−0.883** *	0.317	0.650	0.367	−0.567	−0.567	0.667	−0.550
	*p*	0.**014**	0.999	0.406	0.999	0.784	0.784	0.350	0.875
17:00–19:00 h	Rho	−0.133	0.200	−0.033	0.300	0.650	−0.083	0.000	0.633
	*p*	0.999	0.999	0.999	0.999	0.406	0.999	0.999	0.469
All day									
07:00–19:00 h	Rho	0.167	0.417	0.000	0.283	−0.133	0.667	0.550	−0.167
	*p*	0.999	0.999	0.999	0.999	0.999	0.350	0.875	0.999

*Note:* REMS latency according to AASM criteria; sleep stage 2 (N2), slow wave sleep (SWS, N3) and REMS in minutes within sleep period time; REMS‐ and SWS‐polarity: time relative to sleep onset. Illuminance: median for indicated time slots measured vertical at eye. Spearman's rho (*n* = 9). Bold values: significant correlations. *p* values were adjusted for multiple comparisons (i.e., the seven time frames) using the Bonferroni‐Holm correction.

* Represents significance.

### 3.3 REMS

REMS duration in minutes yielded no significant correlations with the preceding light exposure. However, illuminance showed an association with shorter REMS latency (during midday [11:00–13:00 h]: Rho = 0.817, *p* = 0.049; Table 3). In addition, lower illuminance at midday from 11:00 to 13:00 h was associated with earlier REMS polarity (Rho = 0.817, *p* = 0.049).

### 3.4 Non‐REMS

Lower afternoon illuminance from 15:00 to 17:00 h was associated with more N2 duration (Rho = −0.883, *p* = 0.014, Table 3).

## 4 Discussion

Data confirm and extend previous reports on surprisingly low light levels perceived by people during habitual urban winter days (Espiritu et al. 1994; Savides et al. 1986; Scheuermaier, Laffan, and Duffy 2010 270). In our study, 70% of the day was spent with less than 80 lx perceived vertical at the eyes and 33% with even less than 20 lx. Light levels were extremely low in the afternoon, when about half of the time light levels did not exceed 20 lx and illuminance levels were hardly ever higher than 500 lx. These lower values compared to those previously reported may be attributed to (1) the location of the sensors vertically at the eye as opposed to the wrist, (2) differences in local latitudes and (3) the use of medians instead of means. Mean illumination values did not show correlations with sleep parameters, but median values did. Of course, at an average level of about 100 lx, a few minutes of gazing at the blue sky with 10.000 lx increases the mean values to much higher levels than the median. Data suggest that these short periods of high illumination do not influence subsequent sleep whereas longer periods do.

**FIGURE 3:**
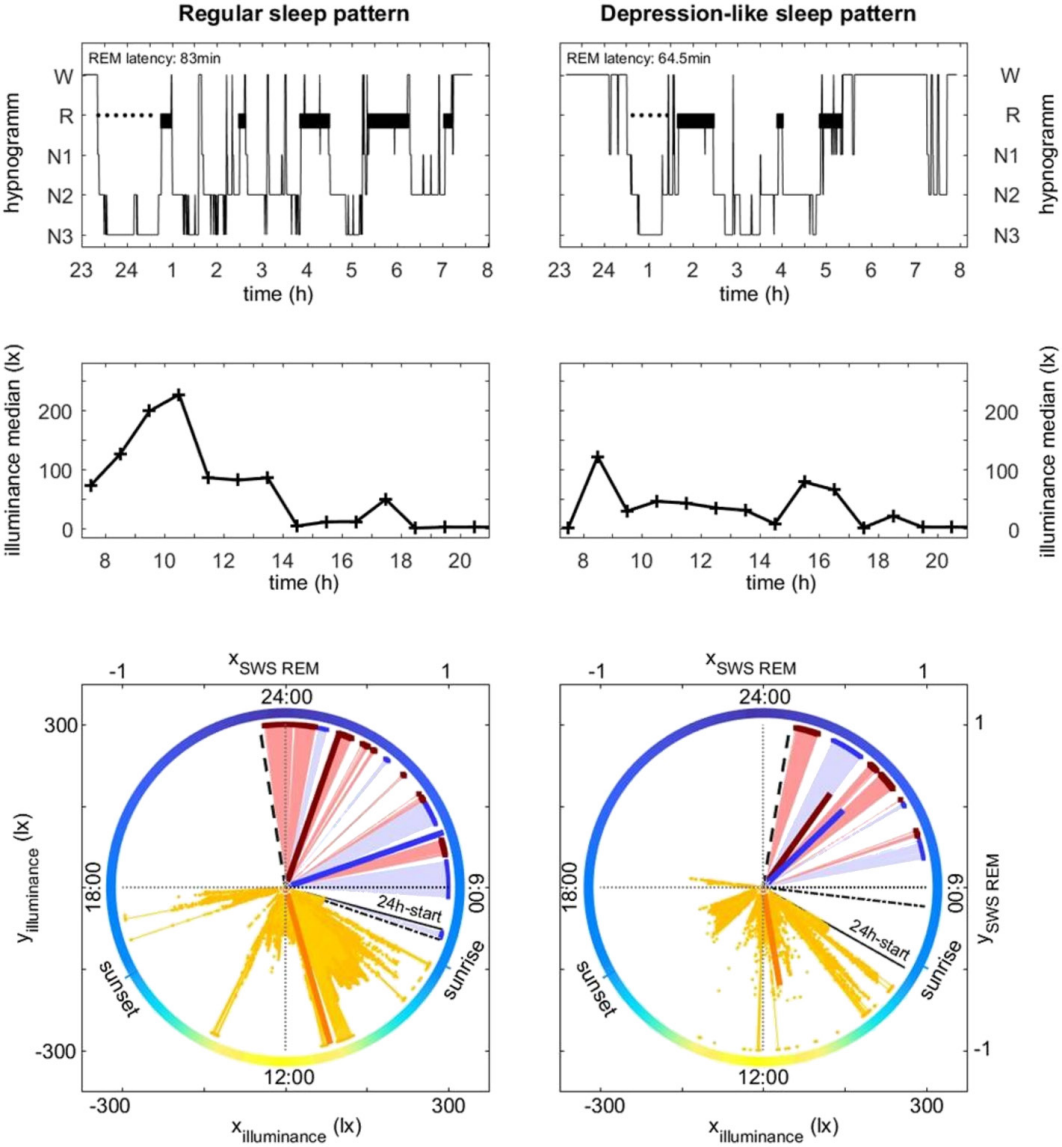
Two extreme examples: ‘regular’ versus ‘depression‐like’ hypnogram and daytime illuminance. *Upper panels*: hypnograms; W = wake, R = REM sleep, N1, N2, N3 = NREM stages N1, N2, N3; N3 is synonymous with SWS; REM latency = elapsed time between sleep onset and the first epoch of REM sleep. *Middle panels*: prior day median illuminance (lx) per hour at eye level. *Bottom panels*: summed polarity vectors for REMS (dark blue) and SWS (dark red); these are obtained by considering every 30‐s epoch within the sleep period time as being a vector with standard length 1 and angle expressed by clock time (24:00 h format) and then adding up the vectors of similar respective sleep stages (REMS, light blue; SWS, light red). The angle of the summed vector thus represents a weighted sum, ‘polarity’, having a specific clock time. Its length expresses the strength of the polarity, with low values indicating low presence and/or considerable temporal dispersion of the respective sleep stage. A similar approach has been used to depict the summed polarity vector (dark orange) of the varying daytime illuminance levels (light orange, with lengths corresponding to the respective illuminances in lx, instead of standard length 1). For representation purposes, the strength of the sleep summed vectors in the figure has been divided by 200 and that of illuminance by 10,000. ——— = start of the experimental day; −−−− = sleep onset; −·−·−· = end of sleep. Note the low daytime illuminance associated with the depression‐like sleep pattern, indicated by problem to initiate sleep, early morning awakening, shortened REMS latency, reduced REMS polarity and shift of SWS to later sleep times (nearly overlapping angles and low strengths of the summed polarity vectors for REM and SWS).

Our participants spent most of their time with indoor low light intensities (i.e., < 200 lx), as opposed to natural daylight outdoors (i.e., 50.000–100.000 lx). Even within the range of such low light intensities, we found significant associations with sleep architecture. REMS latency and REMS distribution over the night (REMS polarity) are well known to be driven or modulated by the circadian timing system (Bes et al. 1996; Dijk and Czeisler 1995; Endo et al. 1981; Khalsa et al. 2002). The lower the light level to which participants were exposed during the midday, the shorter the REMS latency was and the more REMS polarity tended to shift towards the beginning of the night. These associations are well illustrated with two extreme examples of one participant with a regular sleep pattern having been exposed to higher illuminance on the day prior to sleep, and another participant with a depression‐like sleep pattern (Figure 3—problem to initiate sleep, early morning awakening, shortened REMS latency, reduced REMS polarity and shift of SWS to later sleep times) having been exposed to lower illuminance on the day prior to sleep. Describing REM polarity across the night by simply summing vectors in a polar coordinate system might be a promising method to allocate sleep stage REM a centroid (a prevalence time point, e.g., at the beginning or end of the night) and describe its distribution in just two single parameters (time and strength).

If one assumes that our data reflect the usual light situation that urban people are exposed to over the long‐term in winter, then the question of adaptation arises. Studies have found that light history during one prior week affected melatonin suppression (Chang, Scheer, and Czeisler 2011; Hébert, Martin, and Eastman 2002). Spending an Antarctic winter in the absence of natural daylight affected the pupil light sensitivity for months after daylight returned in spring (Kawasaki et al. 2018). Thus, there appears to be a slow and long‐lasting adaptation of the physiology to habitual light levels. Adapted to such a longer period of mainly low light intensities, the physiology may be most sensitive to differences within that range. In agreement with this hypothesis, we previously showed in wake‐EEG that participants adapted to low light levels were sensitive to changes of light spectra at low light intensities, but not to changes in light intensities (de Zeeuw et al. 2019). These dynamics in the low‐light state may be referred to as *Living in Biological Darkness*. By optimizing light characteristics (i.e., light intensity, spectrum, timing, duration and history), circadian responses and sleep may be strengthened (Vetter et al. 2022). But what constitutes optimal daytime light exposure is still an unanswered question (Münch et al. 2020), although studies have shown positive effects of bright daytime lighting on sleep duration and subjective sleep quality (Boubekri et al. 2014; Wams et al. 2017). A systematic review found that while bright light in the morning or evening could shift sleep to earlier or later times respectively, studies showed mixed effects of bright light on other objectively measured sleep parameters such as the number of awakenings and sleep efficiency (Dautovich et al. 2019).

The question arises, what the possible consequences of a long‐term Living in Biological Darkness might be. Our data suggest time‐ and intensity‐dependent associations of light exposure with some of those sleep parameters of the following night that have been suggested as biological markers of depression: REM latency and REM distribution (Kupfer 1976; Kupfer, Ulrich, et al. 1984). Correspondingly, closing the triangle of light, sleep and depression, other studies show the detrimental effects of deficient light conditions on mood and depressive symptoms (Wallace‐Guy et al. 2002) and efficiency of light treatment in major depression (Kripke et al. 1992; Tuunainen, Kripke, and Endo 2004). One mechanism that has been proposed to explain the antidepressant effects of light has been its phase‐shifting effects (Ackermann et al. 2009; Lewy et al. 1980). An alternative hypothesis for (1) sleep changes in depression and (2) effects of light on seasonal depression is a reduced amplitude of the circadian system (Czeisler et al. 1989; Rosenthal and Wehr 1992; Schulz and Lund 1985). Although the concept of circadian amplitude is still controversial, it would explain our data with respect to the ability of light to unfold its effects on REMS regulation.

Since the ‘depression‐like’ sleep pattern was ultimately determined to be nonspecific, one may question its significance. Our data showing that healthy subjects create a depression‐like sleep pattern after a few days of low‐light without any clinical sign of depression suggest a rather ‘healthy’ response without pathophysiological meaning—at least for the short term. Maybe, the development of this sleep pattern in patients with mental illnesses is secondary to the retreat from social life into biological darkness at home.

### 4.1 Limitations of Our Study

Light exposure in our sample was measured in illuminance only, giving no information on spectral composition of the light received. Thus, the mixture of natural and artificial light in our sample was not controlled for. As rods, cones and melanopsin contribute differently to the pathways of non‐visual light effects, this can have influence on brain activity such as sleep. The sample size was small, and data were not normal‐distributed, so we could only perform nonparametric tests. On the other hand, it is impressive that such a small group of healthy young subjects already shows the hypothesized changes in sleep architecture.

## 5 Conclusions

During about four million years of evolution, our ancestors were exposed to natural day light in the range of 3.000 lx (cloudy winter sky) to 100.000 lx (bright sunny sky). Only since the last 150 years, humans have used electrical lighting with light levels—suggested by the data presented here—that seem to be extremely low. Most fascinating is that human physiology appears to adapt and respond differently to such low levels, which is suggested by the association of timing and intensity of habitual daylight with most circadian parameters in sleep architecture. Patients suffering from mental illnesses are likely to spend more time alone and indoors. Our finding of the association between low light levels and changes in sleep architecture that were once suspected to represent biomarkers of affective/mental illness may contribute to a better understanding of some of the mechanisms behind it. It also could explain the missing specificity of changes in sleep architecture once found in mental disorders.

## Author Contributions

**Claudia Nowozin:** conceptualization (equal), data curation (equal), formal analysis (equal), investigation (equal), methodology (equal), visualization (equal), writing – original draft (equal). **Amely Wahnschaffe:** conceptualization (equal), data curation (equal), formal analysis (equal), investigation (equal), methodology (equal), visualization (equal), writing – original draft (equal). **Jan de Zeeuw:** data curation (equal), formal analysis (equal), methodology (equal). **Alexandra Papakonstantinou:** formal analysis (equal), methodology (equal), visualization (equal), writing – original draft (supporting). **Andrea Rodenbeck:** conceptualization (equal), funding acquisition (equal), supervision (equal). **Sven Hädel:** conceptualization (equal), investigation (equal), methodology (equal), software (equal). **Frederik Bes:** formal analysis (equal), methodology (equal). **Dieter Kunz:** conceptualization (equal), funding acquisition (lead), methodology (equal), resources (lead), supervision (lead), writing – original draft (equal).

## Conflicts of Interest

The authors declare no conflicts of interest.

### Peer Review

The peer review history for this article is available at https://www.webofscience.com/api/gateway/wos/peer‐review/10.1111/ejn.16647.

## Data Availability

Data are available upon reasonable request from the corresponding author.
